# The effect of thermal aging on the color matching of Class III and Class V restorations with universal resin composites in various-shade human teeth: an instrumental and visual evaluation

**DOI:** 10.1007/s00784-025-06439-6

**Published:** 2025-06-26

**Authors:** Seyit Bilal Ozdemir, Busra Ozdemir

**Affiliations:** Faculty of Dentistry Department of Restorative Dentistry, Giresun University, Mumcular St & No:1, Giresun, 28100 Turkey

**Keywords:** Esthetics, Color, Resin composite, Spectrophotometry

## Abstract

**Objectives:**

This in vitro study aimed to assess the color matching of two single-shade and one group-shade universal composite resins for Class III and Class V restorations in extracted human teeth of different shades and evaluate the impact of thermal aging on these restorations using both instrumental and visual methods.

**Materials and Methods:**

Forty-five extracted human central incisors in a range of Vita Classical shades (A1, A2 and A3) were used. Three composite resins (Zenchroma (ZC), Charisma Diamond One (CDO), and Neo Spectra ST (NEO)) were tested. Each tooth received both Class III and Class V restorations (2 mm depth and 4 mm diameter) with the same composite resin. Color measurements were made immediately after restoration, 24 h, and after thermal aging using an intraoral spectrophotometer. Color differences (ΔE_00_) were calculated using the CIEDE2000 formula. Visual assessments were scored from 0 (perfect fit) to 4 (significant mismatch) by three dental specialist. Data were analyzed using IBM SPSS V23. A Generalized Linear Model (GLM) was used to assess main and interaction effects, and Tukey’s test was applied for multiple comparisons. A significance level of *p* < 0.05 was considered.

**Results:**

NEO showed statistically significantly lower ΔE₀₀ values (2.45 ± 1.29) compared to the other composite resins (*p* < 0.001). Class III restorations (3.41 ± 1.48) exhibited lower ΔE₀₀ values than Class V restorations (4.56 ± 2.23, *p* < 0.001). ΔE₀₀ values decreased after aging (*p* = 0.007).

**Conclusion:**

Class III restorations demonstrated better color matching than Class V restorations in both visual and instrumental evaluations. The group-shade composite resin showed better color matching compared to the single-shade composite resins. Although visual matching improved over time, some instrumentally measured color differences remained above the clinically acceptable threshold (ΔE₀₀>1.8).

**Clinical relevance:**

Clinicians should consider both composite type and cavity configuration when selecting materials for aesthetic restorations.

## Introduction

The color compatibility, stability, and interactions of composite resins are essential factors when assessing the clinical performance of restorations [1]. Advancements in filler technology have significantly improved the physical, mechanical, and esthetic properties of resin composites, making them widely preferred for both anterior and posterior restorations [2]. Composite resins are currently marketed according to the Vita Classical shade guide (VITA Zahnfabrik, Germany) [3]. Multi shade composite resins are available in individual shades that correspond to each shade in the Vita Classical system. In contrast, group-shade composite resins include fewer shades, each designed to match multiple Vita Classical shades [4]. To eliminate the need for shade selection, single-shade composite resins have been developed [5]. These single-shade universal composite resins are designed to blend with all 16 Vita Classical shades using only one universal shade [4]. They are formulated without additional dyes or pigments [6]. Their ability to blend with surrounding dental tissues is attributed to the chameleon effect [7]. 

A major limitation of composite resin restorations is their tendency to stain, discolor, and lose surface gloss over time, particularly as a result of aging within the oral cavity [8]. Discoloration plays a significant role in restoration failure, especially in anterior teeth where esthetics are critica [9]. This condition often necessitates restoration replacement [10]. To simulate the color changes that may occur in restorative materials in the oral environment, various in vitro methods are employed [11]. These include aging protocols such as immersion in staining solutions and thermocycling, which simulate oral aging conditions [12]. 

For a successful esthetic outcome, the color of the restoration must be indistinguishable from that of the surrounding tooth structure. However, shade selection remains a complex process influenced by environmental and operator-related variables [1]. The polychromatic nature of natural teeth further complicates shade matching [2]. In clinical practice, color evaluation can be performed using both visual and instrumental methods, with visual assessment being the most commonly applied. Although inherently subjective, visual analysis plays a decisive role in patient satisfaction and acceptance of the restoration [13]. Previous studies have shown that visual color perception in dentistry may be influenced by factors such as the clinician’s experience and training [14]. Furthermore, lighter and less saturated shades have been reported to exhibit superior color blending [4, 15]. The restoration site and cavity configuration have been reported to influence smile esthetics in various ways [16]. Studies on color matching in the literature have investigated Class I [1, 4], Class III [17] Class IV [18] and Class V [16] cavity configurations. A recent study assessed shade adjustment in Class I and Class V cavities using visual evaluation on acrylic teeth. Restorations placed on anterior teeth, as well as those on buccal and interproximal surfaces, tend to be more visible and are said to have a greater influence on esthetic outcomes [16]. Because Class III and Class V restorations are located in the anterior region, their ability to blend with natural dentition is of particular importance. Color-matching studies involving extracted human teeth are well documented in the literature [7, 19, 20]. However, evidence regarding the effect of thermal aging on color matching remains limited [18, 21]. Studies are needed to clarify the behavior of different single-shade composites and to compare their performance across different commercial brands [18]. 

The aim of this study was to compare the color matching performance of two single-shade and a group-shade universal composite resins in Class III and Class V restorations on various shade human teeth, using both visual and instrumental methods, and to provide useful clinical insights. Additionally, this study aimed to evaluate the impact of thermal aging on the color matching of these restorations. The null hypothesis was that the universal resin composite type, cavity configuration, and thermal aging would not cause a difference in color matching.

## Materials and methods

### Ethics statement and tooth selection criteria

This study was approved by the Ethics Committee for Non-Drug and Non-Medical Device Research of Necmettin Erbakan University Faculty of Dentistry (2024/506). The inclusion criteria for the study required the buccal surface to be intact, free of discoloration, and without caries or any previous restorations Extracted human incisors were selected by performing shade measurements on the middle third of the labial surface using an intraoral spectrophotometer (VITA Easyshade V, VITA Zahnfabrik, Bäd Sackingen, Germany). Following shade verification, a total of 45 teeth with A1, A2, and A3 shades were included in the study. The teeth were evenly distributed across shade categories (*n* = 15 for each). Each shade group was then randomly and equally assigned to the three composite resin groups.

### Sample size calculation

The sample size was determined using G*Power, with a preliminary study establishing an effect size of 0.42. Based on this effect size, with a Type I error of 0.05 and a statistical power of 0.85, the required sample size was calculated as 45 samples. This calculation was based on three composite resin groups (ZC, CDO, and NEO) and two cavity configurations (Class III and Class V), resulting in six experimental subgroups.

### Experimental groups

This study included two single-shade universal composite resins (Zenchroma (ZC), President Dental, Germany, and Charisma Diamond One (CDO), Kulzer, Germany) and one group-shade universal composite resin (Neo Spectra ST (NEO), Dentsply Sirona, USA). These composite resins were selected to evaluate the color matching of materials with color adjustment potential in extracted human teeth. The compositions of the composite resins used in the study are presented in Table 1.

**Table 1:** Properties of composite resins used in the study

Material	Manufacturer	Lot Number	Type	Monomer	Filler Composition/Size	Filler w/V%	Code
Zenchroma	President Dental, Germany	2,023,001,245	Microhybrid	UDMA Bis-GMA TEMDMA	Glass powder, silicon dioxide inorganic filler / (0.005–3.0 μm)	75/53	ZC
Charisma Diamond One	Kulzer, Germany	M010028	Nanohybrid	UDMA TCD-DI HEA TEGDMA	Barium Aluminium Boro Fluor Silicate Glass / (5–20 μm)	81/64	CDO
Neo Spectra ST HV A1,A2,A3	Dentsply Sirona, USA	21,110,000,808 21,110,000,874 2,103,001,162	Nanohybrid	Bis(4-methyl-phenyl)iodonium hexafluorophosphate	Spherical, pre-polymerized SphereTECfillers, Methacrilate-modified polysiloxane barium glass, and ytterbium flüoride/ ( 3–7 μm)	79/61	NEO

### Specimen Preparation protocol

**Fig. 1:**
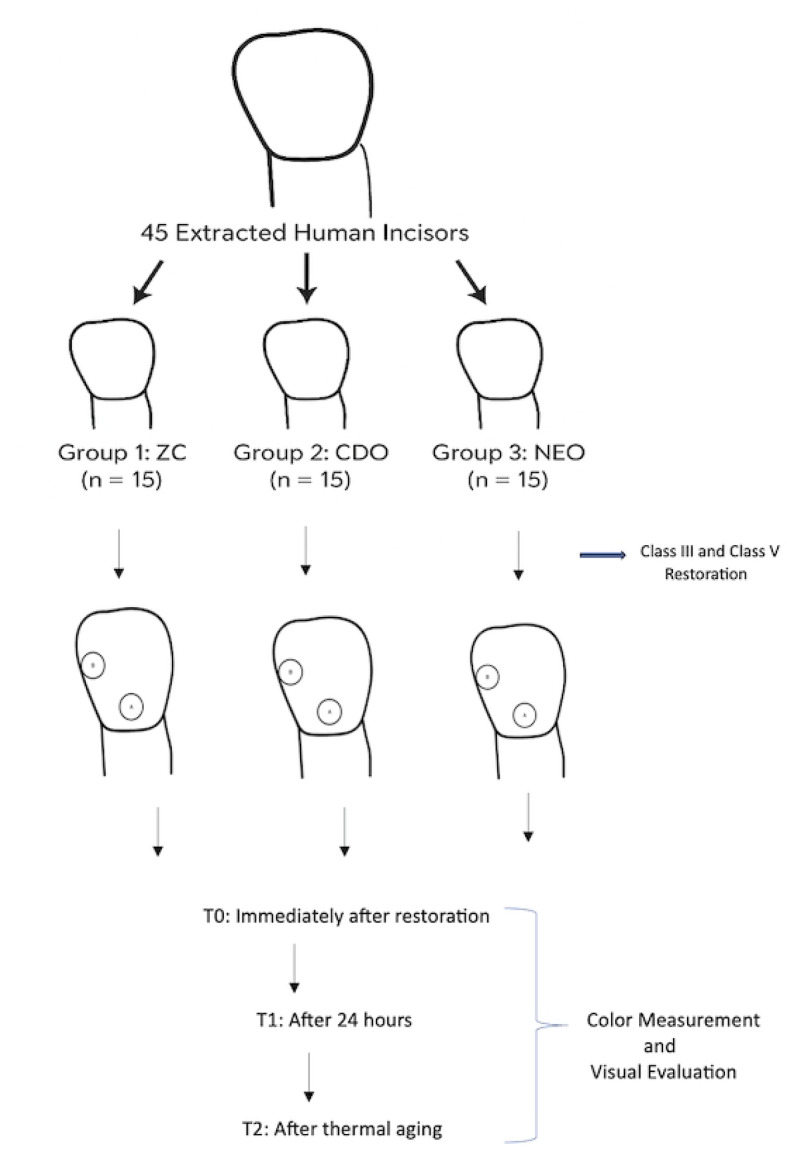
Flowchart of the study

To ensure consistency in the size of the restorations, all cavities were prepared using a silicone guide mold that controlled the cavity dimensions. The location for each preparation was determined using a millimeter probe. For Class V preparations, the midpoint of the mesiodistal dimension was identified 1 mm above the cementoenamel junction. For Class III preparations, the mesial middle third was selected. The silicone guide-mold was positioned at the predefined points, and all cavity preparations were performed by a single experienced operator with over 10 years of clinical experience. The cavities were prepared to a depth of 2 mm and a diameter of 4 mm using a #1014 diamond bur (Kg Sorensen, Brazil). No bevel was incorporated into the preparation process. After cavity preparation, the depth and diameter were verified using a millimeter probe and caliper. Class III and Class V cavities were located on different regions of the same tooth. No shade selection was required for the single-shade universal composite resins. For the group-shade universal composite resin, the appropriate shade was chosen according to the pre-verified tooth shade. Specifically, A1-shade teeth were restored using A1 composite resin, A2-shade teeth with A2 resin, and A3-shade teeth with A3 resin. A comprehensive flowchart of the study is presented in Fig. 1.

### Restoration procedures

After preparation, the cavities were treated with the bonding agent Clearfil S3 Universal Bond (Kuraray Noritake Dental, Tokyo, Japan) according to the manufacturer’s instructions. Polymerization was performed with a Woodpecker LED light device (Woodpecker Medical Instrument Co., Guilin, China) at 1000 mW/cm² for 10 s per cavity. The composite resins were placed into the cavities in a single increment using mylar strip polymerization was performed with a Woodpecker LED light device (Woodpecker Medical Instrument Co., Guilin, China) at 1000 mW/cm² for 20 s per cavity. Restorations were then completed as described below. The samples were then polished by the same operator using fine and super-fine aluminum oxide-coated polishing disks (Sof-Lex Disks, 3 M ESPE) for a total of 30 s.

### Thermal aging procedure

It has been reported that approximately 10.000 thermal cycles correspond to one year of clinical function. Therefore, all samples were subjected to aging through 10,000 cycles in a thermal cycling unit (Thermocycler SD Mechatronic, Feldkirchen-Westerham, Germany). As recommended in ISO/TR 11405:1994, each bath cycle involved a 30-second immersion at temperatures ranging from 5 °C to 55 °C, with a 10-second transfer time between baths [22]. 

### Instrumental evaluation

**Fig. 2:**
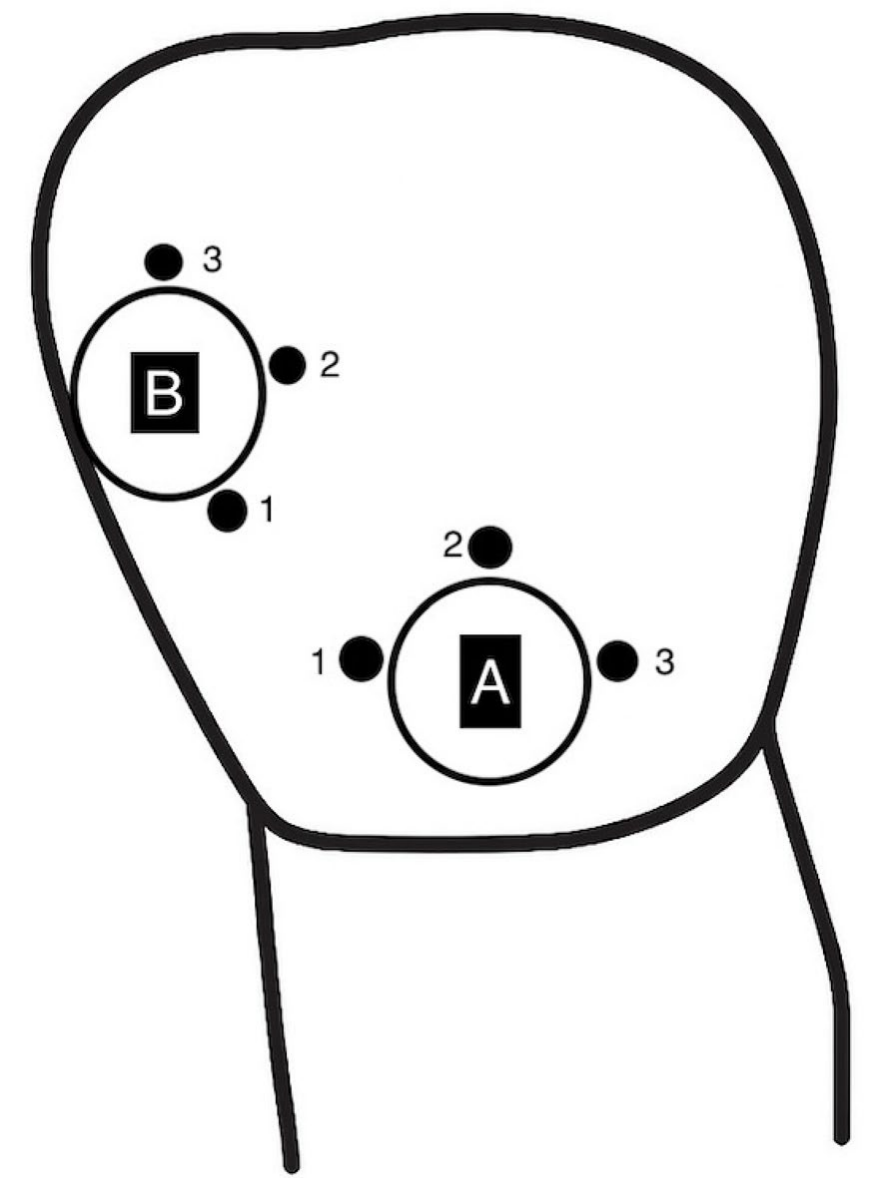
Class V restorations are indicated by symbol A, and Class III restorations are indicated by symbol B. Color was measured using a spectrophotometer from three different points on the tooth surface surrounding the restoration and once directly over the restoration itself

Each tooth was placed on a gray background with the labial surface facing upward before the color measurement. The color differences between the restoration and the surrounding tooth tissue were analyzed. Color measurements were performed using an intraoral spectrophotometer (VITA Easyshade V, VITA Zahnfabrik, Bäd Sackingen, Germany) from three different points of the surrounding tooth tissue and from the top surface of the restoration. L, a, and b values were recorded. A schematic representation of the color measurement regions are presented in Fig. 2. The light probe of the device was positioned perpendicular to the surfaces of the restoration and the tooth. The device was calibrated between measurements. Color measurements were repeated immediately after restoration, after 24 h, and after thermal aging. Samples were stored in distilled water except during the color measurements. The color difference (ΔE₀₀) values were calculated in Excel (Microsoft, USA) using the CIEDE2000 formula provided by Sharma. For each restoration, the mean L*, a*, and b* values obtained from the three measurement points on the surrounding tooth structure were compared with the values from the restoration surface. In this context, ΔL′, ΔC′, and ΔH′ represent the differences in lightness, chroma, and hue between the two compared samples. Weighting functions denoted as S_L, S_C, and S_H were assigned to the lightness, chroma, and hue components, respectively. Moreover, K_L, K_C, and K_H are parametric factors that are adjusted according to various viewing conditions. The parametric factors of the formula were set to 1 [23].

$$\eqalign{& {\rm{\Delta }}{{\rm{E}}_{{\rm{00}}}} \cr& {\rm{ = }}\,\sqrt {{{{\rm{(}}{{{\rm{\Delta }}{{\rm{L}}^{\rm{'}}}} \over {{{\rm{K}}_{\rm{L}}}{{\rm{S}}_{\rm{L}}}}}{\rm{)}}}^{\rm{2}}}{\rm{ + }}\,{{{\rm{(}}{{{\rm{\Delta }}{{\rm{C}}^{\rm{'}}}} \over {{{\rm{K}}_{\rm{C}}}{{\rm{S}}_{\rm{C}}}}}{\rm{)}}}^{{\rm{2}}\,}}\,{{{\rm{(}}{{{\rm{\Delta }}{{\rm{H}}^{\rm{'}}}} \over {{{\rm{K}}_{\rm{H}}}{{\rm{S}}_{\rm{H}}}}}{\rm{)}}}^{{\rm{2}}\,}}{\rm{ + }}\,{{\rm{R}}_{\rm{T}}}{{{\rm{(}}{{{\rm{\Delta }}{{\rm{C}}^{\rm{'}}}} \over {{{\rm{K}}_{\rm{C}}}{{\rm{S}}_{\rm{C}}}}}{\rm{)}}}^{\rm{2}}}\,{\rm{(}}{{{\rm{\Delta }}{{\rm{H}}^{\rm{'}}}} \over {{{\rm{K}}_{\rm{H}}}{{\rm{S}}_{\rm{H}}}}}{\rm{)}}} \cr} $$

In this study, the perceptibility and clinical acceptability thresholds for CIEDE2000 were set at ΔE₀₀ > 0.8 units and ΔE₀₀ ≤ 1.8 units, respectively, based on a 50:50% perceptibility and acceptability model [19]. 

### Visual assessment

Three dental specialist were selected, demonstrating excellent color differentiation ability, according to ISO/TR 28642:2016. The Ishihara test for color blindness was used to assess color deficiency in all participants. For visual evaluation, a product tent with a D65 light source (6500 K) was used under a 0°/45° viewing geometry. The visual evaluation was carried out with the teeth placed against a neutral gray background in a random and blind order, and they were observed from a distance of 25 cm. The color differences between each tooth and its restoration were rated on a scale from 0 to 4, based on a scale from a previous study; where ‘0’ indicated perfect match, ‘1’ very good match, ‘2’ not a very good match (borderline mismatch), ‘3’ obvious mismatch, and ‘4’ significant (obvious) mismatch [24]. Visual assessments were performed immediately after restoration, 24 h, and after thermal aging. Class III and Class V restorations on each tooth were evaluated separately. Prior to visual assessment, the evaluators were calibrated by providing instructions regarding the duration of evaluation and the possible response options. They were also notified that each tooth contained two restorations in different regions. Evaluators were instructed to assess the color matching between each restoration and the surrounding tooth structure. During the assessments, the evaluators were blinded to both the type of composite resin and the timing of the evaluation.

### Statistical analysis

Statistical analyses were performed using IBM SPSS V23, Minitab V14, and Jamovi V2.3.28. The normality of the data was assessed using the Shapiro-Wilk test. Since the data were normally distributed, a Generalized Linear Model (GLM) was used to evaluate the main effects of composite type, cavity configuration, and time on ΔE₀₀ values, as well as their interaction effects. When statistically significant differences were identified, Tukey’s post-hoc test was employed for pairwise multiple comparisons to determine which groups differed significantly. Additionally, Fleiss’ Kappa statistic was used to assess interobserver agreement among the three evaluators at each visual assessment time point (immediately after restoration, after 24 h, and after thermal aging). Kappa values were interpreted as follows: ≤ 0.20 = slight agreement, 0.21–0.40 = fair agreement, 0.41–0.60 = moderate agreement, 0.61–0.80 = substantial agreement, and 0.81–1.00 = almost perfect agreement. A significance level of *p* < 0.05 was considered for all statistical tests.

## Results

**Fig. 3:**
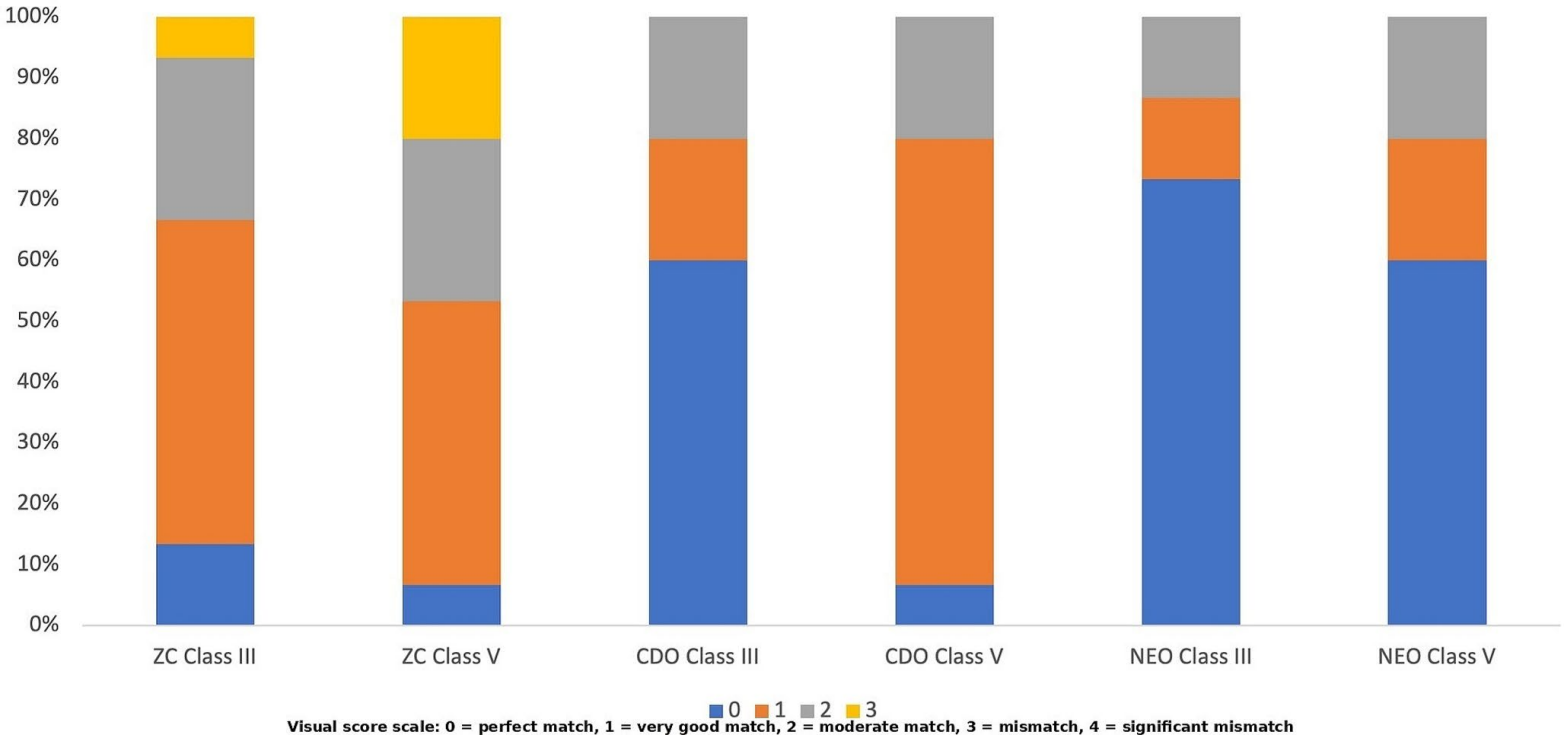
Color match scores of the groups immediately after time (ZC = Zenchroma, CDO = Charisma Diamond One, NEO = Neo Spectra ST)

**Fig. 4:**
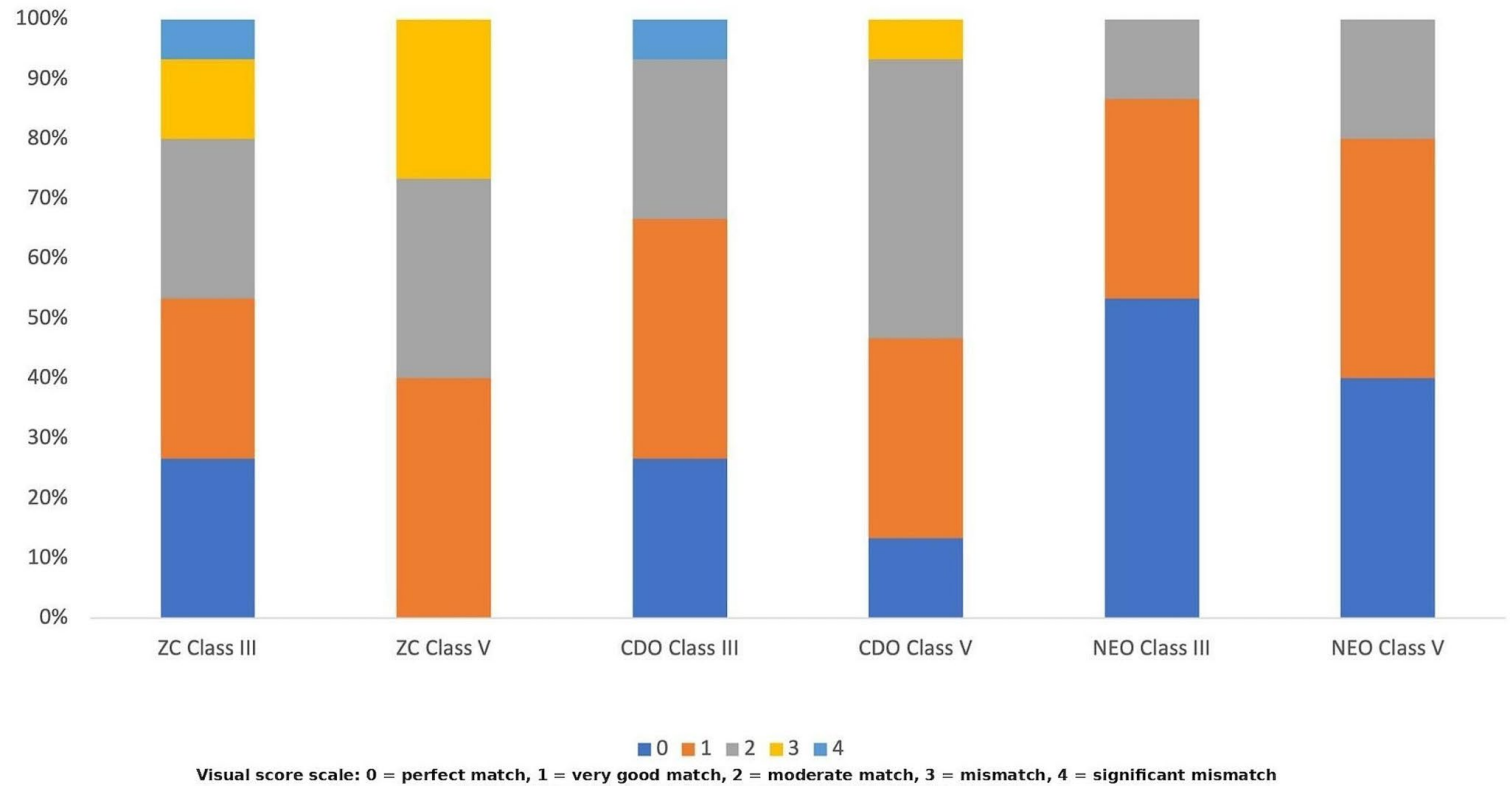
Color match scores of the groups 24 h later (ZC = Zenchroma, CDO = Charisma Diamond One, NEO = Neo Spectra ST)

**Fig. 5:**
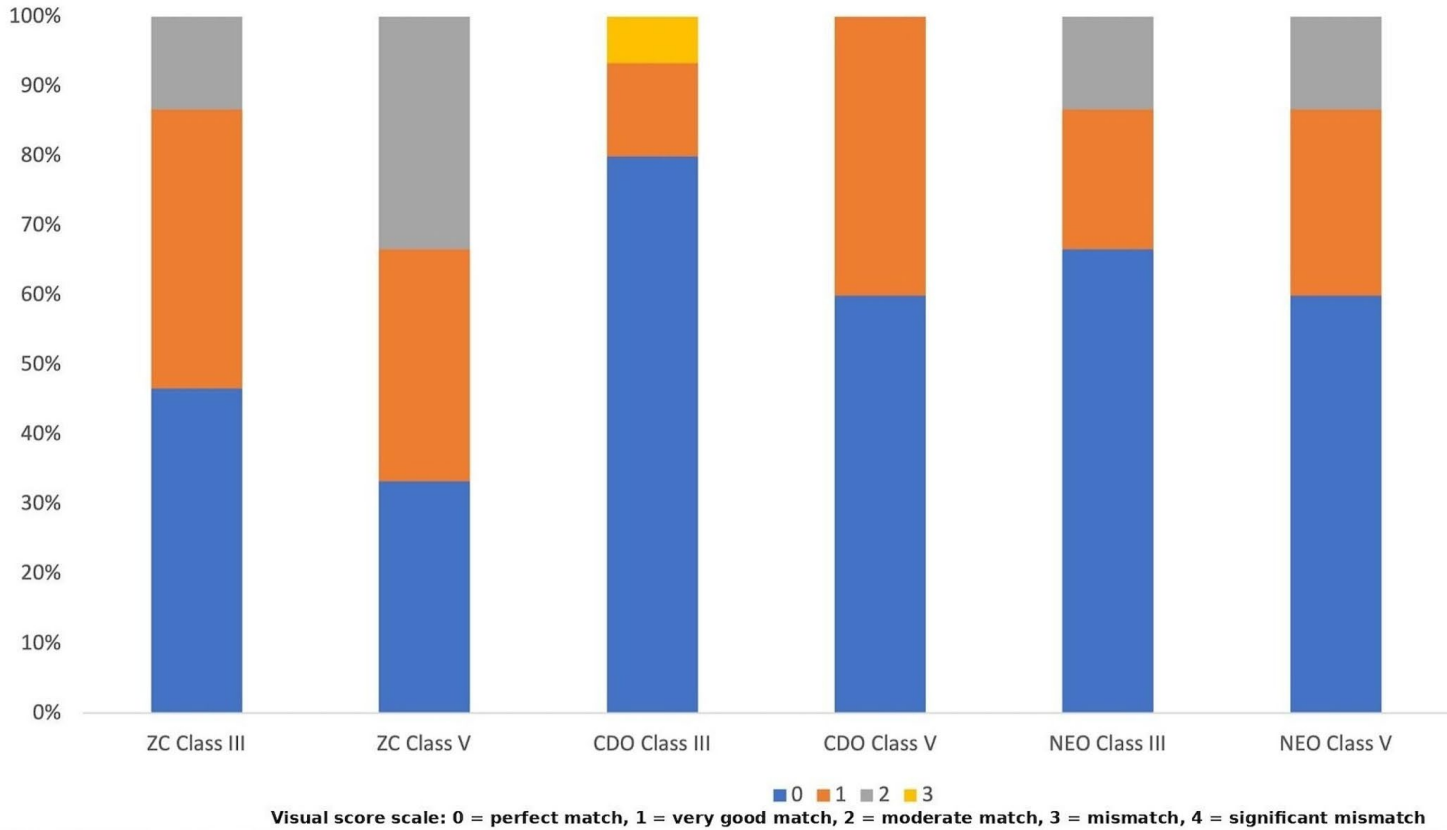
Color match scores of the groups after aging (ZC = Zenchroma, CDO = Charisma Diamond One, NEO = Neo Spectra ST)

The comparison of ΔE₀₀ values based on composite type, cavity configuration, and time revealed statistically significant differences. The effect of composite type on ΔE₀₀ values was statistically significant (*p* < 0.001). NEO (2.45 ± 1.29^b^) exhibited significantly lower ΔE₀₀ values than the other resin composites (Table 2). The effect of cavity configuration on ΔE₀₀ values was also statistically significant (*p* < 0.001). Class III cavities demonstrated lower ΔE₀₀ values compared to Class V cavities (Table 2). The effect of time on ΔE₀₀ values was also statistically significant (*p* = 0.007). ΔE₀₀ values significantly decreased after aging compared to those measured immediately after restoration **(**Table 2). The interaction between composite type and cavity configuration on ΔE_00_ values was statistically significant (*p* = 0.017). Among all restorations, the highest significant ΔE_00_ values were observed for the CDO Class V restoration. With the exception of one value (NEO-Class III at 24 h), all mean ΔE₀₀ values observed in this study exceeded the clinical acceptability threshold of 1.8. The agreement between evaluators was assessed using Fleiss’ Kappa statistic. The inter-rater agreement was weak both immediately after restoration (Kappa = 0.375; *p* < 0.001) and after aging (Kappa = 0.237; *p* = 0.003), while no agreement was found at 24 h (*p* = 0.867). Visual evaluations of restorations performed immediately after restoration, after 24 h, and after aging are shown in Figs. 3 and 4, and 5, respectively. No ratings of 4 (significant mismatch) were observed for immediate after restoration and after aging evaluations (Figs. 3 and 5). The percentage of 0 (perfect match) ratings was higher after aging compared to immediately after restoration (Figs. 3 and 5). When visual evaluations at all time points were examined, Class III restorations in each composite group showed a higher percentage of 0 (perfect match) ratings compared to Class V restorations (Figs. 3 and 4, and 5). Although this result was consistent with the instrumental evaluation, the measured color differences exceeded the clinical acceptability threshold. Nevertheless, visual assessments still included 0 (perfect match) scores. This result, suggests that visual perception of color match can still be favorable even when ΔE₀₀ values exceed the clinical threshold.

**Table 2:** Multiple comparison results of ΔE_00_ values by composite, cavity and time

Cavity	Time	Composite			Total
		ZC	CDO	NEO	
Class III	Imdt after	3.96 ± 1.31	4.50 ± 0.73	2.07 ± 1.22	3.51 ± 1.49^B^
	24 h	4.44 ± 1.58	3.89 ± 1.05	1.67 ± 1.03	3.34 ± 1.70^B^
	After aging	4.44 ± 1.30	3.54 ± 0.26	2.13 ± 1.05	3.37 ± 1.34^B^
	Total	4.28 ± 1.32^BC^	3.98 ± 0.81^BC^	1.96 ± 1.04^D^	3.41 ± 1.48
Class V	Imdt after	4.74 ± 1.28	8.44 ± 2.25	3.86 ± 1.42	5.68 ± 2.59^A^
	24 h	4.75 ± 1.22	5.40 ± 1.31	3.15 ± 0.96	4.43 ± 1.46^AB^
	After aging	4 ± 0.9	4.82 ± 2.69	1.83 ± 0.91	3.55 ± 2.05^B^
	Total	4.50 ± 1.12^B^	6.22 ± 2.59^A^	2.95 ± 1.35^CD^	4.56 ± 2.23
Total	Imdt after	4.35 ± 1,29	6.47 ± 2.61	2.97 ± 1.56	4.6 ± 2.35^a^
	24 h	4.6 ± 1.34	4.65 ± 1.37	2.41 ± 1.22	3.88 ± 1.65^ab^
	After aging	4.22 ± 1.08	4.18 ± 1.92	1.98 ± 0.94	3.46 ± 1.71^b^
	Total	4.39 ± 1.21^a^	5.1 ± 2.20^a^	2.45 ± 1.29^b^	3.98 ± 1.97

^A−D^ There is no difference between interactions with the same letter. ^a−b^ There is no difference between groups with the same letter. Mean ± Standard deviation

## Discussion

Achieving accurate color matching is essential for the effectiveness of composite resin restorations [25]. This factor is linked to how acceptable the restoration is for both dental professionals and patients [26]. Achieving an ideal color match depends on multiple interrelated factors [27]. Thermal cycling simulates the aging process and thermal fluctuations in the oral cavity. It has been suggested that 10,000 thermal cycles can be considered equivalent to one year of clinical usage [28]. In our study, a thermal aging procedure equivalent to one year was performed. Based on the results of the current study, composite type, cavity configuration, and time were found to have a significant effect on ΔE_00_ values, thus the null hypothesis of the study was rejected.

Single-shade universal composite resins were claimed to reduce color mismatch [29]. However, studies reported that multi-shade universal resin composites provided better color matching compared to single-shade resin composites [20, 24]. In line with these results, in this study, the NEO group shade universal composite exhibited the lowest ΔE_00_ values. Various factors can influence the color matching of composite resins, including filler size and content, the composition of the resin matrix, the size of the restoration, the layering technique, and the shade and brand of the composite resin itself [30]. Additionally, the material composition is important, as composites often contain color pigments and metal oxides [4]. The fact that NEO exhibited the lowest ΔE₀₀ values among all tested composites was expected, given its group-shade formulation. This material contains color pigments and is designed to match the full range of Vita Classical shades using only a few ‘cloud’ tones. This may explain why the NEO group-shade composite resin provided better color matching. However, some studies have suggested that both single-shade and group-shade universal resin composites exhibit similar color matching behavior [16, 31]. The findings of this study highlight the importance of selecting composite resins based on their optical properties and color matching potential. In clinical practice, group-shade composites with pigment-based formulations may provide superior esthetic outcomes, particularly in anterior regions. The highest ΔE_00_ values among all restorations were exhibited by the CDO composite group. The resin matrix content affects the translucency of composite resins. Resin composites containing Bisphenol A diglycidyl methacrylate (Bis-GMA) have been reported to be more translucent than those without Bis-GMA [30]. Translucency was found to show a positive correlation with the blending effect [32]. The color matching in this study was correlated with the filler composition. While ZC contains Bis-GMA in its matrix, CDO does not include it. Additionally, the hydrophilic nature of TEGDMA in the CDO structure and its ability to absorb water between polymer chains, resulting in a more heterogeneous matrix, may have increased color differences [11]. Reis et al. [33] stated that the structure of the material and the amount of organic matrix filler are important factors affecting color change in resin composites. On the other hand, the smaller particle size found in resin composites may explain their lower susceptibility to coloration. The presence of smaller filler particles is associated with lower susceptibility to surface roughness and coloration compared to other resin composites with larger filler particles [34]. CDO has the largest filler particle size among the resin composites. This could explain its higher susceptibility to coloration and the higher ΔE_00_ values it exhibited. The performance of single-shade composites may vary depending on the brand and formulation. Additionally, in a study that evaluated the color adjustment potential (CAP) of various single-shade resin composites on human teeth of different shades using both instrumental and visual methods, it was reported that CDO did not differ significantly from other single-shade composites and exhibited an acceptable CAP [7]. 

The chameleon effect in composite resins was affected by the size of the restoration [35]. Paravina et al. [32], concluded that color matching in restorations improved as cavity size decreased and the translucency of the filling material increased. Therefore, in this study, the sizes of Class III and Class V restorations were kept the same. In this study, Class III restorations exhibited lower ΔE_00_ values compared to Class V restorations. Ruiz-López et al. [16] reported that Class I resin composite restorations exhibited superior color matching compared to Class V restorations. This difference was attributed to the way light is reflected by smoother surfaces, such as buccal, and scattered by irregular surfaces, such as occlusal. There is a general color transition from the most saturated cervical region to the incisal region of the teeth [2]. It was also noted that the color of composite resin is influenced by its surrounding environment This situation [4]. This may have resulted in lower color blending and higher ΔE₀₀ values in Class V restorations compared to Class III restorations. The lower ΔE₀₀ values observed in Class III restorations compared to Class V restorations suggest that cavity location and surrounding tooth morphology can influence esthetic results. These findings suggested clinicians should be cautious when using single-shade composites in cervical cavities.

Thermal cycling is one of the aging methods developed to simulate the physical effects caused by the long-term clinical use of restorative materials in a short period and under stable conditions [22]. It has been noted that immersion aging results in significantly greater color changes compared to thermal cycling. This can be attributed to the static and continuous nature of immersion, in contrast to the dynamic and intermittent effect of thermal cycling [12]. ΔE_00_ values immediately after restoration were significantly higher than those after aging. It has been reported that allowing the teeth to dry makes them appear whiter [36]. This may explain the higher ΔE_00_ values immediately after restoration. It was also reported that Easyshade’s performance was equal to or better than that of the dentists, with the agreement between visual and instrumental findings rated as good to excellent [37]. Parallel to the decrease in ΔE₀₀ values observed after aging compared to immediately after restoration in instrumental evaluation, an increase in the percentage of 0 (perfect match) ratings was observed in the resin composite groups during visual assessments after aging (Fig. 5). This may be attributed to the translucency property of the resin composites used in this study. During the polymerization process, the refractive index difference between the filler particles and the matrix reduces, leading to an increase in translucency. This could improve color matching over time [38]. It has been noted that the ability of single-shade resin composites to reflect the color of surrounding tooth tissues improves over time, which may result in better color matching as time progresses [20]. ​ For these reasons, the reduction in ΔE_00_ values after thermal aging can be explained. The improvement in color matching of the tested composite resins after aging suggests that the esthetic integration of restorations may improve over time in clinical practice. In a study conducted by Mohammadipour et al., the single-shade composite Omnichroma showed a significant reduction in ΔE_00_, particularly against a B1 background, leading to improved color matching after aging [39]. In a study conducted by Santana et al. [18], the tested monochromatic composite resins demonstrated better adaptation after thermocycling. These findings are consistent with the results of our study. However, there are also studies reporting that thermocycling negatively affects shade adjustment [9, 40].

Different methods such as visual, photographic, and instrumental analyses have been used in the literature to evaluate color matching [4, 24]. In instrumental methods, a spectrophotometer is employed. Spectrophotometers may provide more successful clinical outcomes in color selection compared to other methods [41]. They evaluate the intensity of light reflected from a surface and its spectral characteristics [42]. In order to improve correlation with visual perception, ISO and CIE now suggest applying the CIEDE 2000 color difference formula (ISO/CIE 11664-6:2014) [43, 44]. In this study, ΔE_00_ values were calculated using CIEDE2000. According to the CIEDE2000 formula, color differences were interpreted using the 50:50% perceptibility threshold (PT) and acceptability threshold (AT), which are set at 0.8 and 1.8, respectively, as reported in previous study [44]. With the exception of one case (NEO-Class III at 24 h), all mean ΔE₀₀ values observed in this study exceeded the clinical acceptability threshold of 1.8. In contrast to the findings of the present study, it has been reported that single-shade composite restorations placed in cervical cavities exhibited comparable and clinically acceptable ΔE₀₀ values [21]. The natural dentition is characterized by a polychromatic appearance, partial translucency, and a curved anatomical form—all of which influence how light is reflected and dispersed. These variables may impact the optical behavior of resin composites and affect the outcomes of their evaluation in in vitro settings using instrumental measurement techniques [45]. While the visual method is inherently subjective, the visual evaluation of color matching or mismatch often plays a significant role in the overall acceptance of the restoration by the patient [16]. It has been reported that experienced dentists demonstrate significantly higher visual color matching, independent of color guides and lighting conditions [46]. Therefore, visual evaluation was performed by three different specialist dentists in this study. In this study, both instrumental and visual assessments were utilized. In the visual evaluations, restorations were rated using a five-point scale: 0 corresponded to a perfect match (ΔE₀₀ ≤ 0.8), 1 to a very good match (ΔE₀₀ > 0.8 and ≤ 1.8), 2 to an acceptable match (ΔE₀₀ > 1.8 and ≤ 3.6), 3 to a slight mismatch (ΔE₀₀ > 3.6 and ≤ 5.4), and 4 to a significant mismatch (ΔE₀₀ > 5.4), as previously defined in the literature [13, 32]. According to the visual evaluation results, Class III restorations in each composite group showed better color matching compared to Class V restorations. Even when ΔE₀₀ values exceed the clinical threshold, the visual perception of color matching may still be favorable. This situation may be explained by various factors that influence the perception of color matching, such as the shape and location of the tooth within the dental arch, reflections from the oral cavity, the amount of residual tooth structure, and the characteristics of the surrounding hard and soft tissues [4]. Instrumental color mismatches may not always correspond to patient dissatisfaction or indicate restoration failure. Similar to our findings, it has been reported that although visual analysis showed clinically acceptable color matching, spectrophotometric evaluation initially revealed high ΔE values. However, in that study, ΔE values became clinically acceptable after thermal aging [18]. This difference may be attributed to the use of the CIELab color system, which is an older and less detailed version compared to CIEDE2000.

“The current study, being an in vitro study involving extracted human teeth, is unable to directly simulate oral conditions. The findings of this study may be affected by various factors associated with both the tooth structure and the resin composite being assessed. Longer-term aging studies may be needed. Although trained observers were used, visual assessment may be subjective. Clinical studies are recommended to validate the results of this in vitro study, with specific focus on different cavity configurations, adhesive strategies, and long-term color stability under real oral conditions.

## Conclusion

The group-shade composite resin (NEO) demonstrated better color matching than the single-shade composites. Class III restorations showed better color matching than Class V restorations in both visual and instrumental assessments. Additionally, ΔE₀₀ values decreased after thermal aging compared to immediately after restoration. Although perfect match (score 0) ratings were observed in visual evaluations at all time points, the instrumentally measured ΔE₀₀ values still exceeded the clinically acceptable threshold.

## Data Availability

The dataset generated and/or analyzed during the current study are available from the corresponding author on reasonable request.
